# Maillard Reaction Crosslinked Alginate-Albumin Scaffolds for Enhanced Fenofibrate Delivery to the Retina: A Promising Strategy to Treat RPE-Related Dysfunction

**DOI:** 10.3390/pharmaceutics15051330

**Published:** 2023-04-24

**Authors:** Maria Abedin Zadeh, Raid G. Alany, Leila Satarian, Amin Shavandi, Mohamed Abdullah Almousa, Steve Brocchini, Mouhamad Khoder

**Affiliations:** 1 Drug Discovery, Delivery and Patient Care (DDDPC) Theme, School of Life Sciences, Pharmacy and Chemistry, Kingston University London, Kingston Upon Thames KT1 2EE, UK; r.alany@kingston.ac.uk; 2 UCL School of Pharmacy, University College London, London WC1N 1AX, UK; s.brocchini@ucl.ac.uk; 3 School of Pharmacy, The University of Auckland, Auckland 1010, New Zealand; 4 Department of Stem Cells and Developmental Biology, Cell Science Research Center, Royan Institute for Stem Cell Biology and Technology, ACECR, Tehran 1665659911, Iran; l.satarian@royan-rc.ac.ir; 5 3BIO-BioMatter, École Polytechnique de Bruxelles, Université Libre de Bruxelles (ULB), Avenue F.D. Roosevelt, 50-CP 165/61, 1050 Brussels, Belgium; amin.shavandi@ulb.be; 6 Duba General Hospital, Saudi Ministry of Health, Duba 49313, Saudi Arabia; maalmousa@gov.sa

**Keywords:** alginate, BSA, Maillard reaction, age-related-macular degeneration, drug release, fenofibrate, retinal cells

## Abstract

There are limited treatments currently available for retinal diseases such as age-related macular degeneration (AMD). Cell-based therapy holds great promise in treating these degenerative diseases. Three-dimensional (3D) polymeric scaffolds have gained attention for tissue restoration by mimicking the native extracellular matrix (ECM). The scaffolds can deliver therapeutic agents to the retina, potentially overcoming current treatment limitations and minimizing secondary complications. In the present study, 3D scaffolds made up of alginate and bovine serum albumin (BSA) containing fenofibrate (FNB) were prepared by freeze-drying technique. The incorporation of BSA enhanced the scaffold porosity due to its foamability, and the Maillard reaction increased crosslinking degree between ALG with BSA resulting in a robust scaffold with thicker pore walls with a compression modulus of 13.08 KPa suitable for retinal regeneration. Compared with ALG and ALG-BSA physical mixture scaffolds, ALG-BSA conjugated scaffolds had higher FNB loading capacity, slower release of FNB in the simulated vitreous humour and less swelling in water and buffers, and better cell viability and distribution when tested with ARPE-19 cells. These results suggest that ALG-BSA MR conjugate scaffolds may be a promising option for implantable scaffolds for drug delivery and retinal disease treatment.

## 1. Introduction

Retinal pigment epithelium (RPE) is a monolayer of nonregenerative cells that is vital for the retinal functional integrity and vision cycle. Located between the neural retina and choroid, RPE plays an essential role in transporting nutrients and waste products, absorbing stray light, phagocytosing shed photoreceptor membranes and secreting growth factors [1]. Ageing, diabetes, and smoking are important factors leading to RPE dysfunction and subsequent retinal degeneration diseases such as age-related macular degeneration (AMD) and Diabetic Retinopathies (DR) which are major causes of visual impairment and vision loss [2]. Globally, the number of AMD cases increases rapidly and is expected to reach about 288 million by 2040 [3].

The intravitreal injection of steroids and anti-vascular endothelial growth factor (anti-VEGF) remains preferred over other conventional treatments, such as laser and photodynamic therapy, for retinal disease management [2,4], especially in the early stages of the disease. However, the main drawback of the intravitreal treatment is the need for repetitive injections due to the poor ocular bioavailability of drugs after topical administration [5]. Fenofibrate (FNB) is a fibric acid derivative used to treat abnormal blood lipid levels. FNB acts as a prodrug that is rapidly hydrolysed in vivo to form its active fenofibric acid metabolite. FNB came into medical use in mid-1970s and was first marketed in the United States in 1988 (Tricor^®^, Abbott Laboratories) [6]. FNB showed promising therapeutic effects in clinical trials for the management of retinal diseases such as DR and neovascular AMD [7]. When compared with anti-VEGF, FNB is advantageous given its low-cost, fewer side effects, and neuroprotective activity [7,8]. However, the use of FNB for dysfunctional RPE treatment is challenging due to its very hydrophobic nature (log *P* = 5.24) and poor water solubility. RPE transplantation was introduced as an alternative and effective way to replace damaged RPE cells with healthy ones [9]. However, the implementation of this approach is hindered by the shortage of tissue donors, heavy surgical intervention, and risks of serious post-surgical infections and transplanted cell rejection [1]. 

Recently, implantable polymeric scaffolds capable of providing structural support for retinal cell growth and proliferation have gained considerable attention for retinal regenerative treatment [10]. The same scaffolds can be used to locally deliver active therapeutics, such as growth factors and active pharmaceutical ingredients, at the damaged retinal sites to enhance cell growth and reduce side effects [11]. Ideally, scaffolds should display physicochemical and biological properties that mimic those of native tissues. This includes good biocompatibility, controllable biodegradability, high porosity and drug loading capacity, and sufficient mechanical stability during and post-implantation [1]. The fundamental features of scaffolds are dependent on the nature of the comprising polymers and composition, crosslinker types (either chemical or physical), and methods of fabrication [1,12]. Due to their safety, biodegradability, chemical versatility, low cost, and processability, naturally occurring polymers, such as sodium alginate (ALG), have been explored to fabricate scaffolds for tissue regenerative applications [1,13]. In the presence of divalent cations, such as calcium, ALG solution forms gel via ionotropic crosslinking, resulting in a 3D hydrogel structure [14]. This unique feature was successfully and widely used to produce ALG scaffolds for different pharmaceutical and biomedical applications [15,16,17]. However, the hydrophilic nature of ALG leads to fast swelling and poor mechanical properties, hence an uncontrollable drug release behaviour [15]. A promising approach to improve ALG scaffold’s performance is to modulate the crosslinking density between ALG chains using chemical crosslinking alongside ionotropic crosslinking [18]. However, chemical crosslinking requires reagents and solvents that often have toxic side effects [19].

Maillard reaction is a natural non-enzymatic process during which covalent bonds are formed between carbonyl groups of reducing sugar and the free amino groups of amino acids, peptides, and proteins [20]. This reaction is spontaneous, thus does not necessitate the use of any toxic chemicals or organic solvents [21]. Furthermore, Maillard reaction products possess antioxidant, anti-inflammatory, and radical scavenging properties which are beneficial for tissue regeneration and development [22,23]. Bovine serum albumin (BSA) is a naturally occurring biocompatible protein that plays a structural support role in cell proliferation and growth and has recently gained considerable attention in the development of scaffolds for tissue repair and regenerative medicine [24,25]. In a previous study, we showed that when ALG is conjugated with BSA via Maillard reaction, the beads produced using the ALG-BSA Maillard product possessed superior water resistance, viscosity, foamability, and capability to control the drug release compared with that of pristine ALG [20].

ALG reinforced by the Maillard reaction has not been previously used to fabricate scaffolds for ocular applications. The aim of this study is to design and develop FNB-loaded scaffolds based on ALG-BSA Maillard conjugate, which can provide a suitable environment for RPE cells to grow, migrate, and produce their own extracellular matrix (ECM). The use of these scaffolds as FNB delivery system is assessed. Morphological and mechanical characteristics as well as the porosity of obtained scaffolds are investigated and the scaffolds swelling, drug loading capacity, and drug release profile are established. Finally, biological investigations are performed to assess the scaffold’s biodegradability and biocompatibility with seeded ARPE-19 cells (a spontaneously arising retinal pigment epithelium (RPE) cell line) on ALG-based scaffolds.

## 2. Materials and Methods

### 2.1. Materials

ALG (medium molecular weight: 120,000–200,000 g/mol; M/G ratio: 0.8), BSA, FNB, calcium chloride (CaCl_2_), sodium chloride (NaCl), sodium lauryl sulfate (SLS), phosphate-buffered saline (PBS), 2-(4-(2-hydroxyethyl)-1-piperazinyl) ethane sulphonic acid (HEPES), potassium bromide (KBr), sodium citrate, and acetonitrile (I) were all purchased from Sigma-Aldrich chemicals (Dorset, UK). For the in vitro cell study, ARPE-19 cells, a spontaneously immortalised human RPE cell line, were obtained from ATCC (Manassas, VA, USA). Dulbecco’s Modified Eagle Medium: F12 (DMEM/F12), fetal bovine serum (FBS), penicillin/streptomycin, trypan blue and trypsin/ethylenediaminetetraacetic, DAPI (4′,6-diamidino-2-phenylindole), calcein-AM, propidium iodide (PI) were purchased from Gibco (Invitrogen, CA, USA). The MTS assay kit was provided by Promega (Madison, WI, USA). Milli-Q distilled deionised (DI) water was used for all experiments.

### 2.2. Fabrication of the Scaffolds

Scaffolds were fabricated by a freeze-drying method [26]. Briefly, to prepare ALG solution (1.5% *w*/*v*), 0.75 g of ALG was gradually added and dissolved in 50 mL of DI water. For ALG-BSA scaffolds, of BSA (0.25 g) was added to the obtained ALG solution to make ALG-BSA solution. The ALG and a blended mixture of ALG-BSA solutions were separately poured into a well of 96-well culture plate (150 μL per well), and frozen overnight before being freeze-dried (VirTis Benchtop Pro, SP scientific, Winchester, UK) for 48 h. Obtained freeze-dried scaffolds were then crosslinked with calcium by immersion into a 2% (*w*/*v*) CaCl_2_ solution for 30 min. Crosslinked scaffolds were then freeze-dried for another 48 h to remove the solvent completely. For drug-loaded scaffolds, FNB was added and mixed with the obtained ALG and ALG-BSA solution at a concentration of 0.2% (*w*/*v*). For MR-ALG-BSA scaffolds, obtained ALG and ALG-BSA scaffolds were placed into a desiccator containing an oversaturated KBr solution to control the relative humidity at 79% and incubated for 24 h at 60 °C in an oven (Binder, Germany).

### 2.3. Morphological Characterisation

Scanning electron microscopy (SEM) was used to examine the microstructural properties of the scaffolds. Prior to imaging, specimens’ surface was coated with a conductive layer of sputtered gold, and the electron microscope (Zeiss Evo50, Oxford instrument, Abingdon-on-Thames, UK) was operated at an accelerating voltage of 30 kV under low-vacuum mode. SEM images were employed to determine the average pore size and porosity of scaffolds. At least twelve pores were randomly selected from each scaffold SEM image and analysed using ImageJ software (V 1.8.0), and pore size data were presented as mean ± standard deviation.

### 2.4. Mechanical Properties Testing

Mechanical properties of the dry and rehydrated scaffolds were investigated using a texture analyser (Santam, STM-29, Tehran, Iran) with a 1 n load cell at a crosshead speed of 1 mm/min. To simulate the composition of the vitreous humour, scaffold rehydration was performed in HEPES buffer (pH = 7.4 at 37 °C) containing 132 mM NaCl, mimicking the pH and the electrocyte concentration of the vitreous humour [27]. During the experiment, the rehydrated scaffolds remained immersed in the buffer. The compressive stress–strain curves were generated following the methods reported by Wan et al. [28]. The Young’s modulus was calculated as the slope of the stress–strain curve at 10% strain.

### 2.5. Swelling and Degradation Studies

The scaffolds swelling behaviour were evaluated in DI water, PBS, and HEPES-NaCl buffer. Briefly, the scaffold was weighed and placed separately in 10 mL of each medium at 37 ± 0.5 °C. At specific intervals, the scaffold was removed from the swelling medium, and the excess medium was carefully removed with filter paper and weighed. The swelling ratio was calculated using the following equation [29]:

(1) $$swelling\;ratio(\%)=\left[\frac{Ws-Wi}{Wi}\right]\times 100\%$$

where *W_i_* is the initial weight of the scaffold, and *W_s_* is the weight of swollen scaffold.

The biodegradability of scaffolds was assessed by immersion in DMEM/10% FBS culture medium at 37 ± 0.5 °C to provide conditions similar to those of the cell culture studies. Scaffolds were then incubated in a shaker incubator (ThermoFisher Scientific, Swindon, UK) that was operated at 100 rpm. On days 1, 3, 7, and 14, scaffolds were removed from the medium, frozen, and freeze-dried for 48 h, and the weight loss of scaffolds was calculated using the following equation:

(2) $$Degradation(\%)=\left[\frac{Wd-Wr}{Wd}\right]\times 100$$

where *W_d_* is the initial weight of the scaffold and *W_r_* is the weight of the degraded scaffold.

### 2.6. Drug Loading and Release Studies

To investigate the loading capacity and release of FNB, the saturation solubility of FNB was first determined in HEPES-NaCl buffer solution (pH 7.4) with and without SLS (25 mM). Briefly, an excess amount of FNB (10 mg) was added to the (10 mL) HEPES-NaCl buffer and shaken at the speed of 100 rpm at 37 °C for 48 h. Samples were then centrifuged for 15 min and filtered through a polytetrafluoroethylene (PTFE) syringe-driven filter (average pore size = 0.45 μm). The FNB solubility was determined using HPLC. An Agilent Infinity II HPLC system (Agilent, Waldbronn, Germany) was employed. The flow rate was adjusted at 1 mL/min, and the injection volume was 10 µL. Chromatographic separation was achieved at room temperature using a C18 column (4.6mm × 150 mm, C18, 5 μm) (Phenomenex SphereClone), and a UV detector was set at a (λmax) 286 nm. The mobile phase consisted of 90:10 *v*/*v* acetonitrile/HPLC grade water [30,31].

#### 2.6.1. Loading Capacity Study

To determine the loading capacity of drug-loaded formulations, one scaffold was dispersed in 1 mL of a 55 mM sodium citrate solution at 37 °C, resulting in the full release of FNB. The resulting suspension was filtered and diluted with 9 mL of HEPES-NaCl buffer, which contained SLS at a concentration of 25 mM. The FNB content was then measured using HPLC, and the loading capacity was calculated using the following equation [32]:

(3) $$Loading\;capacity\;\left(\%\right)=\left[\frac{L}{Lt}\right]\times 100$$

where *L* and *L_t_* are the measured and theoretical amounts of fenofibrate loaded in the scaffold, respectively.

#### 2.6.2. Drug Release Study

The FNB-loaded scaffolds were soaked separately in 10 mL of HEPES-NaCl buffer containing SLS (25 mM) and placed in a shaking incubator at 100 rpm and 37 °C. At specific time intervals (1, 2, 4, 6, and 8 h, and then 2, 3, 4, and 6 days), 500 μL of samples were withdrawn from the release medium and replaced with fresh release medium to keep the initial volume constant. The amount of FNB released was determined using the HPLC.

### 2.7. Biological Investigations

#### 2.7.1. Cell Culture and Seeding on Scaffolds

ARPE-19 cells were expanded and maintained in a typical RPE medium containing DMEM supplemented with 10% foetal bovine serum (FBS) and 1% penicillin-streptomycin solution. Cells were used between passages 31 and 41 for the experiments. At 80–90% confluency, cells were subcultured using 0.05 trypsin-EDTA. The collected cells were centrifuged at 1500 rpm for 5 min and resuspended in a new medium.

Prior to the cell seeding process, scaffolds were sterilised by immersion in ethanol (70%) for 20 min. The scaffolds were then soaked in the cell culture medium for 30 min and transferred into a well of 48 well-plates. ARPE-19 cells were then counted with a haemocytometer by using the standard trypan blue method and cells in suspension (5 × 10^4^ cells/mL) were seeded on top of the scaffold surface. After 2 h of incubation, fresh culture medium (500 μL) was added to each well of the cell culture well-plate and kept in standard conditions at 95% humidity, 5% CO_2_, and 37 °C. The medium was changed every other day intervals during the experiments.

#### 2.7.2. MTS-Based Cytotoxicity Assay

To determine the viability of the ARPE-19 cells, MTS assay was performed [33]. Briefly, after the incubation of the cell-seeded scaffolds for 1, 3, 7, and 14 days, the media was removed, and the scaffolds were rinsed with PBS. MTS solution (1:5 dilution in culture medium) was added to the seeded scaffolds and placed in a dark room for 3 h at 37 °C and 5% CO_2_. The optical density of each sample was measured at 490 nm using a UV spectrophotometer microplate reader (FlexStation3 Multi-Mode, Molecular Devices, San Jose, CA, USA). The negative controls were cell culture medium without cells.

#### 2.7.3. Analysis of ARPE-19 Cells-Scaffold Interactions

To visualise the presence of ARPE-19 cells in the scaffolds, the DAPI staining method was used. The cells were first seeded onto each scaffold following the same procedure described above. At day 14, scaffolds were washed twice with PBS before being stained with 200 μL of DAPI solution. Cells-seeded scaffolds were then incubated for 15 min in a dark room at 37 °C. The samples were washed with PBS three times to remove excess DAPI. To assess cell-scaffold interaction, ARPE-19 cells within scaffolds were stained with a LIVE/DEAD staining solution on day 14 of cell seeding. For this purpose, stock solutions of calcein-AM (3 μM) and PI (2.5 μM) were separately prepared, diluted in PBS, and kept at 4 °C. Immediately before use, a working LIVE/DEAD staining solution was prepared by mixing 100μL of calcein-AM and 100 μL PI. Each scaffold was washed with PBS and stained with the LIVE/DEAD solution. After 15 min of incubation at 37 °C in dark, samples were immediately observed under a confocal microscope (Zeiss, Jena, Germany) to evaluate viable and damaged cells. Samples were excited with 498 nm and with 540 nm laser wavelengths and the emitted fluorescence was detected at 515 nm and 615 nm for calcein-AM and PI detections, respectively [34]. Viable cells were illustrated in green fluorescence by calcein-AM and dead cells were illustrated in red by PI. The control was the blank scaffolds without cells.

#### 2.7.4. Distribution of Cells on the Scaffolds

The distribution of ARPE-19 grown on scaffolds was examined using scanning electron microscopy (Vega, Tescan, Czech Republic). On day 14 of cell seeding, scaffolds were rinsed with PBS and washed with double-deionised water. Scaffolds were then frozen in liquid nitrogen and freeze-dried for 48 h. Gold was sputtered on the scaffolds with fixed cells to form a 15 nm thick layer on the surface with a deposition rate of 1 Å/s, and SEM images were captured.

### 2.8. Statistical Analysis

All experiments were performed at least in triplicate, and results were reported as mean ± standard deviation (SD). Statistical significance was determined using one-way analysis of variance (ANOVA) with post-hoc Tukey’s HSD test. *p*-values < 0.05 indicated statistical significance.

## 3. Results & Discussion

### 3.1. Scaffolds Structural Morphology

**Figure 1:**
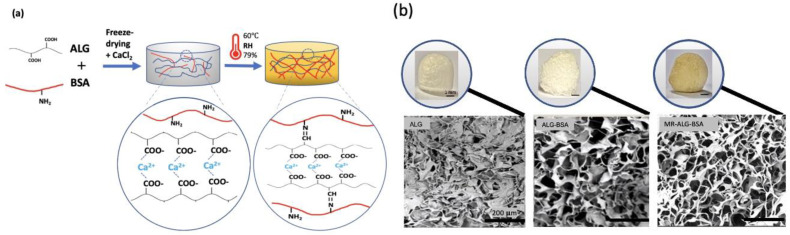

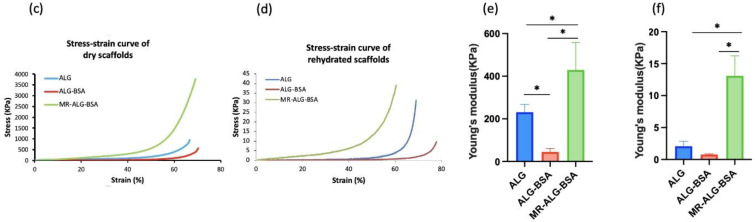
Characteristics of the ALG-based scaffolds; (**a**) schematic illustration of non-treated and MR ALG-based scaffolds, (**b**) the visual (scale bars 1 mm) and SEM (scale bars 200 µm) appearance of ALG, ALG-BSA and MR-ALG-BSA, (**c**) the compressive stress–strain curves of dried and (**d**) rehydrated scaffolds, (**e**) Young’s moduli of dried and (**f**) rehydrated scaffolds (*n* = 3). (* denotes a significant difference, *p* < 0.05).

Maillard reaction is an established method that involves the covalent bonding between the amino group (NH_2_) of a protein and the carbonyl group (C=O) of reducing sugars [20]. The conjugation of ALG with BSA via Maillard reaction is therefore expected to add a chemical crosslinking in the polymeric scaffolds, in addition to the ionotropic crosslinking between ALG carboxylic acid and Ca^+2^ [35]. Maillard reaction can also generate other types of chemical bonds, such as those between amino acids and the various reactive intermediates produced during the reaction. Briefly, in the early stage, the reducing sugar reacts with the NH_2_ groups of the protein to form a Schiff base. This reaction is reversible and can lead to the formation of Amadori products in the intermediate stage. In the intermediate stage, the Amadori products undergo further reactions, resulting in the formation of advanced glycation end products (AGEs). These products are highly reactive and can react with other proteins, lipids, and nucleic acids, leading to the formation of crosslinks. The intermediate stage can also produce reactive carbonyl compounds, such as 3-deoxyglucosone and glyoxal, which can react with the amino groups of proteins to form covalent crosslinks. In the final stage of the Maillard reaction, melanoidins are formed which are brown pigments responsible for the colour of many food products. In this study, the colour of scaffolds subjected to the Maillard reaction changed from white to brownish (Figure 1b), indicating the formation of ALG-BSA melanoidins [36]. Schematic illustrations of non-treated and treated scaffolds are shown in Figure 1a.

Figure 1b illustrates that all scaffolds possessed porous structures of micro-sized pores, with enhanced porosity observed upon the incorporation of BSA. ImageJ software analysis showed that while the average pore size of ALG scaffolds was 53.16 ± 18.04 µm, this significantly increased in the presence of BSA to 110.8 ± 23.9 µm and 107 ± 21.4 µm in ALG-BSA and MR-ALG-BSA scaffolds, respectively. Notably, a previous study reported that an optimal pore diameter to support retinal cells ranged between 100–200 μm [37]. The porosity of ALG scaffolds was initially 39% and has increased after the incorporation of BSA to 51% and 56% for ALG-BSA and MR-ALG-BSA scaffolds, respectively. BSA is known for its foaming capacity [38], leading to the formation of bubbles during the preparation of the scaffold solutions. Upon freeze-drying, air bubbles that acted as porogen turned into pores that increased pores size diameter and porosity in BSA-containing scaffolds [24,39]. Larger pore size obtained after the incorporation of BSA in the scaffold is expected to facilitate the exchange of nutrients and waste products with the surrounding environment and provide a more favourable structure for cell growth compared to ALG scaffolds [40,41]. Furthermore, the MR-ALG-BSA scaffolds exhibited a thicker pore wall with a measurement of 13.33 ± 2.74 µm, which was significantly higher compared to the pore wall thicknesses of ALG and ALG-BSA scaffolds (5.9 ± 1.85 µm and 4.3 ± 1.21 µm, respectively). This higher thickness of MR scaffold pores walls might potentially increase the overall mechanical properties of the scaffolds, as thicker walls can provide better support and resistance to deformation [42].

### 3.2. Mechanical Properties of the Scaffolds

The mechanical properties of fabricated scaffolds were evaluated in dry and rehydrated conditions in HEPES-NaCl. The PBS was not used for rehydration to avoid destabilisation of the Ca-ALG structure [43]. Figure 1c–f shows the compressive stress–strain curves and Young’s modulus of dried and rehydrated scaffolds, respectively. Both the incorporation of BSA in the scaffolds’ formulation and Millard reaction significantly affected the compressive strength of the scaffolds. For non-treated ALG-BSA samples (i.e., did not go through MR reaction), the compressive strength was lower than that of pure ALG scaffold (841.58 KPa and 691.18 KPa respectively). On the other hand, conjugating BSA with ALG reversed its impact, leading to an increase in the compressive strength and young’s modulus to 3913.79 KPa and 465.11 KPa, respectively.

The Maillard reaction results in the formation of covalent bonds between the ALG carboxyl groups and the BSA amino groups, leading to a higher molecular weight product and increased crosslinking [44]. Our results indicated a stiffer structure of MR scaffolds compared to the non-treated ALG-BSA samples. Furthermore, the mechanical strength of scaffolds is influenced by porosity, with larger pore sizes having a negative impact, while thicker pore walls, resulting in smaller pore sizes, improve the strength [41,42].

Since scaffolds are rehydrated before implantation, further investigation was performed on rehydrated scaffolds. Due to water absorption and weakening bonds within the scaffolds structure [45], the Young’s modulus of rehydrated pure ALG scaffolds decreased to 2.07 KPa, as shown in Figure 1f. This result is in agreement with the compressive modulus value of about 3 KPa reported by Wan et al. [28] for ALG scaffolds.

As per dry scaffolds, rehydrated MR-ALG-BSA scaffolds displayed the highest Young’s modulus at 13.08 KPa. Since scaffolds act as a temporary support that must withstand mechanical stress until the tissues are regenerated, MR-ALG-BSA scaffolds are promising, exhibiting substantially enhanced mechanical performance in both dry and wet conditions. It is worth mentioning that the compressive modulus of MR-ALG-BSA (13.08 KPa) falls within the range of the compressive modulus of a healthy retina, which has been reported to be between 10–20 KPa [46,47]. Thus, the MR-ALG-BSA scaffolds are expected to provide an advantageous environment for retinal cell survival, differentiation and phenotypic maintenance.

### 3.3. Scaffolds Swelling, Biodegradation, and Drug Release Studies

- Swelling Studies

The swelling behaviour of scaffolds is a determinant factor that affects mechanical properties upon rehydration, drug release, biodegradation, and cellular activities. While excessive swelling might lead to the collapse of scaffolds and uncontrolled release of drugs, failing to swell could prevent drug release [48]. Similarly, adequate swelling is indispensable for cell migration and the transfer of cell nutrients and metabolites in and out of the matrix. Furthermore, swelling expands the pores and maximises the scaffold’s surface area, allowing for more room for cells to grow [49].

Swelling studies were performed in three different media: water, PBS, and HEPES-NaCl buffer solutions. Up to six hours, blank and FNB-loaded scaffolds showed identical swelling behaviours in all media.

**Figure 2:**
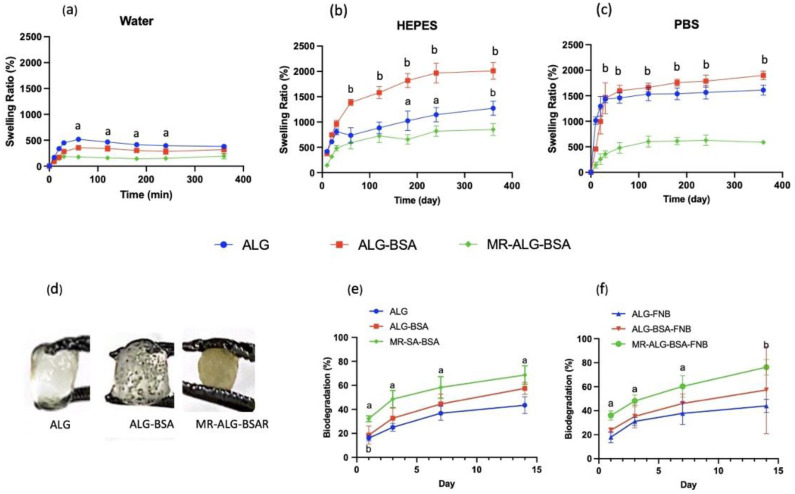
Swelling profiles of ALG, ALG-BSA and MR-ALG-BSA scaffolds in (**a**) water, (**b**) HEPES and (**c**) PBS. (**d**) The visual appearance of scaffolds after rehydration overnight in HEPES-NaCl buffer. The degradation profiles of (**e**) dried and (**f**) rehydrated scaffolds after 1, 3, 7 and 14 days in cell culture medium. (a: *p* < 0.01, b: *p* < 0.001 significant difference between MR scaffold and the other scaffolds) (*n* = 3).

Considering their mechanical properties, the physical addition of BSA (i.e., BSA-ALG scaffold) led to a significant increase in swelling ratio in all media. However, when BSA was conjugated with ALG (i.e., MR-ALG-BSA scaffolds), the swelling ratio significantly dropped (*p* > 0.05). A similar impact of the Maillard reaction on the swelling capacity of alginate gels was previously reported [50]. All scaffolds exhibited a swelling rate that was approximately five-times higher in buffered solutions (PBS and HEPES) compared to water (Figure 2a–c). ALG scaffolds absorb water and undergo swelling due to the abundance of hydrophilic groups such as hydroxyl and carboxyl [51,52]. In buffered solutions, the ionisation of carboxylic groups causes electrical repulsion, which leads to further swelling.

The rate and degree of hydrogel network swelling can be influenced by other factors such as crosslinking density and porosity. In addition, external factors such as the pH, composition and ionic strength of the swelling medium can also affect the extent of swelling behaviour [53]. Contrary to water, PBS and HEPE solutions have the capacity to maintain a stable pH at around 7 [54] which leads to full ionisation of ALG-free carboxylic acid groups (pKa = 4–5). This repulsion amongst the negatively charged carboxylic acid groups causes increased polymer swelling. Buffered media also contain monovalent cations such as Na^+^ and K^+^ that could penetrate the scaffold matrix and displace the Ca^2+^. Since monovalent cations cannot crosslink alginate, the matrix structure relaxes and swells. The Maillard reaction converts ALG carboxylic acid groups into an amide group preventing their ionisation and reducing the overall swelling of the scaffolds. Upon extending the swelling study to 48 h, FNB-loaded scaffolds incubated in the PBS buffer fully disintegrated. However, the scaffolds incubated in water and HEPES buffer did not swell any further and maintained their integrity for up to 72 h. A similar destabilising impact of PBS buffer on Ca-ALG structures was reported [14,43,55], which was attributed to the chelating effect that phosphate ions can have on Ca^2+^ and the subsequent formation of a calcium phosphate precipitate. Figure 2d displays the visual appearance of the swollen scaffolds in the HEPES-NaCl solution after 24 h.

- Biodegradation

To investigate the stability of prepared scaffolds in a cell culture medium, the biodegradation of scaffolds was assessed for 14 days. Scaffolds biodegradation is essential for tissue regeneration; in vivo cell activities, such as distance and signal transduction within the matrix, must be maintained until the cells produce their own EMC [1]. Furthermore, scaffolds biodegradation products are eliminated by the eyes, i.e., there is no need to remove them later on. Figure 2e,f shows the degradation profiles of blank and FNB-loaded scaffolds in a cell culture medium over 14 days. Although there was a gradual loss of mass in all scaffolds as incubation time increased, they remained intact for a period of 14 days. The degradability of scaffolds could be explained by the loss of crosslinking Ca^+2^ due to exchange with non-crosslinking monovalent cations (Na^+^) that are present in cell culture medium. The incorporation of BSA in scaffold formulation resulted in a faster and higher degradation rate than those of ALG scaffolds, suggesting the dissolution and release of BSA. Compared with ALG and ALG-BSA, MR-ALG-BSA scaffolds (blank and loaded with FNB) showed the highest mass loss of about 25–40% in 24 h and about 60–80% (respectively) after 14 days of incubation in the cell culture medium. This could be due to the uncontrolled nature of the Maillard reaction, leading to the production of intermediate compounds such as Amadori products, that could result in such discrepancy [20,56].

During the degradation process, the scaffolds with larger pores and higher surface area to volume ratio (i.e., ALG-BSA and MR-ALG-BSA scaffolds) became further loose as the surrounding aqueous solution came in contact with the internal network structure. This was expected to correlate with the swelling behaviour in buffered media. However, the MR-ALG-BSA scaffolds showed the lowest swelling ratio with the highest degradation rate. A high degradation rate of BSA-containing scaffolds can be advantageous in providing a better environment to generate ECM and promote cell–cell and cell–scaffold interactions. However, the optimal degradation rate, matching the rate of retinal tissue regeneration, is yet to be identified [1].

- Drug Loading and In-Vitro Release Studies:

To avoid any PBS buffer interference with the drug release, FNB release studies were conducted using HEPES buffer. FNB is a lipophilic compound (Log P = 5.24) that is non-ionisable and insoluble in water [57]. To perform the release studies, the solubility of FNB was first established in the release medium (i.e., HEPES buffer) which was found to be practically insoluble (1.35 ± 0.003 µg/L). To allow the free release of FNB into the surrounding medium, SLS (0.025 M) was added to the HEPES buffer. SLS forms micelles that solubilise the hydrophobic drug such as the FNB. The apparent solubility of FNB in the presence of SLS increased to 105 ± 0.02 µg/L. The scaffold loading capacity was measured by fully dissolving drug-loaded scaffolds in sodium citrate solution, allowing the full release of FNB. The effectiveness of sodium citrate as a chelating agent for the dissolution of Ca-ALG scaffolds has been reported in the literature [58,59]. The dissolution process occurred through ion exchange, where the Na^+^ ions in sodium citrate were replaced with the chelated Ca^2+^ ions in the ALG-based scaffolds, leading to the loss of crosslinking and the dissolution of the formulated scaffold [60].

**Figure 3:**
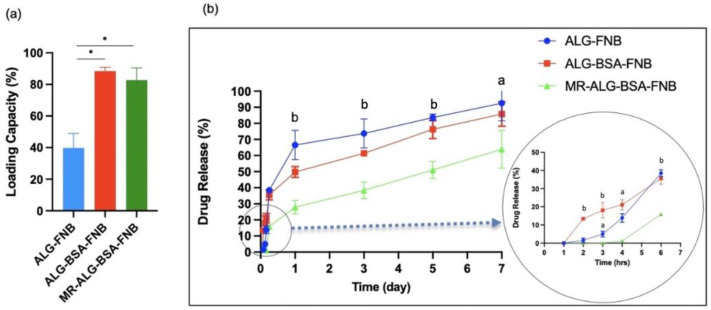
(**a**) drug loading capacity, (**b**) FNB release profiles from ALG, ALG-BSA and MR-ALG-BSA scaffolds in HEPES. (*: *p* < 0.05. a: *p* < 0.01, b: *p* < 0.001 significant difference between MR scaffold and the other scaffolds) (*n* = 3).

Figure 3a shows the FNB loading capacity of scaffolds. ALG, ALG-BSA, and MR-ALG-BSA scaffolds’ loading capacities were 39 ± 2.45%, 88 ± 1.90%, and 82 ± 6.28%, respectively. Previous studies have reported that the highly hydrophilic nature of alginate makes loading a significant amount of hydrophobic drugs rather challenging [61,62]. Interestingly, BSA molecules can bind hydrophobic molecules leading to the formation of soluble complexes, which could explain the enhanced FNB loading capacity of BSA-containing scaffolds [63].

The release profiles of FNB from the ALG, ALG-BSA and MR-BSA-ALG scaffolds are presented in Figure 3b. All scaffolds revealed 1st order release kinetics where the rate of release decreases over time. Both ALG and ALG-BSA scaffolds exhibited initial burst release with approximately 40% of loaded FNB released in the first 6 h and almost 90% released in 6 days. Interestingly, MR-ALG-BSA scaffolds displayed significantly slower release with no release in the first 4 h, about 25% released at 6 h, and almost 60% release within 6 days. The drug release data correlated with the reduced swelling of MR-ALG-BSA scaffolds, which is plausible given that ALG-based systems typically operate through a swelling/diffusion-controlled release mechanism. Khoder et al. also observed similar findings, where ALG-BSA Maillard product beads demonstrated more sustained release than physically mixed ALG-BSA beads in a simulated intestinal medium. This was attributed to the higher molecular weight of the ALG-BSA conjugate, enhanced viscosity, and reduced swelling of the system [20].

### 3.4. In Vitro Biological Investigation with ARPE-19 Cells

- Viability of the ARPE-19 cells within scaffolds

The biocompatibility of ALG scaffolds in retinal regenerative medicine was investigated by culturing ARPE-19 cells on them. This cell line is commonly used for eye research applications due to its convenience and consistency for cell culture studies [64]. MTS assay was used to evaluate the viability of ARPE-19 cells in direct contact with the fabricated scaffolds.

**Figure 4:**
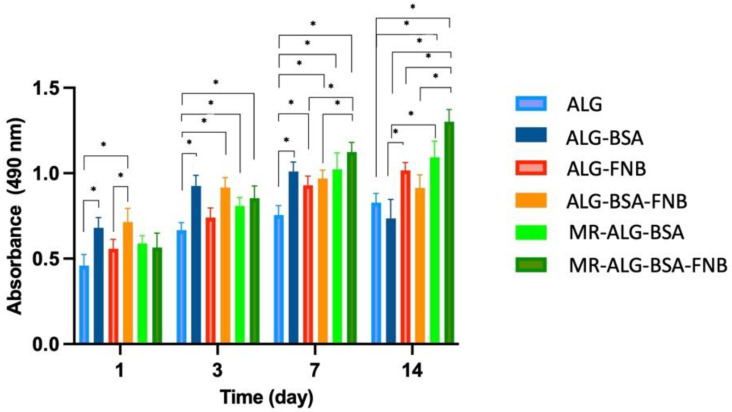
The ARPE-19 cell viability determined by the MTS assay after 1, 3, 7, and 14 days of seeding them seeded on ALG, ALG-BSA, and MR-ALG-BSA scaffolds (*n* = 4). (* denotes a significant difference, *p* < 0.05).

Figure 4 shows the MTS absorbance values at 490 nm of all scaffolds, which are directly proportional to cell viability. All ARPE-19 cells seeded scaffolds showed MTS absorbance values from day 1, which gradually increased over incubation time, indicating the biocompatibility of scaffolds and ARPE-19 cells viability. Our results showed that the cell viability on the ALG scaffold gradually increased through the entire incubation period. Compared with blank ALG scaffolds, both blank ALG-BSA and MR-ALG-BSA scaffolds demonstrated significantly higher ARPE-19 cell viability, notably after day 7, which might be attributed to their larger pore diameter.

For FNB-loaded scaffolds, an initial significant rise in ARPE-19 cell viability was observed after day one for both ALG-FNB and ALG-BSA-FNB scaffolds. The cell viability on MR-ALG-BSA-FNB consistently increased during the entire cell culture period. This could be due to the anti-inflammatory properties of FNB [65]. It was previously reported that FNB protected retinal cell survival by reducing oxidative stress and inflammation, which was attributed to its PPARα role, decreasing the level of inflammatory mediators, including TNF-α, IL-6, and MCP-1 [66].

The viability of cells on the FNB-loaded MR-ALG-BSA scaffold was significantly higher than the other groups on day 14, which might be explained by the consistent sustained release of FNB from MR-ALG-BSA scaffolds over the tested period. Furthermore, ALG and ALG-BSA scaffolds could be depleted from FNB due to the burst and faster FNB release, as opposed to MR scaffolds, hence could not sustain its beneficial effect until day 14.

- In vitro cell–scaffold interaction studies

In regenerative medicine, the interaction of cells with scaffolds is critical to enhancing the secretion and deposition of ECM, which actively facilitates and regulates cellular activities [12]. Cells–scaffold interaction and cell distribution within scaffolds were studied by staining ARPE-19 cells on the scaffolds with fluorescence staining, including DAPI, calcein-AM, and PI. The DAPI staining forms fluorescent blue products upon binding to DNA in the nucleus [67]. Calcein-AM penetrates the cells and stains the cytoplasm of living cells. Once inside the cells, intracellular esterase enzymatically cleaves acetomethoxy derivative to produce a fluorescent green product. The intensity of green fluorescence is directly proportional to the number of viable cells with intact cell membranes [68]. On the other hand, cells with damaged membranes cannot emit green fluorescence. PI is a non-permeant dye which can penetrate the membrane of damaged cells and produce red fluoresce in dead cells [69].

**Figure 5:**
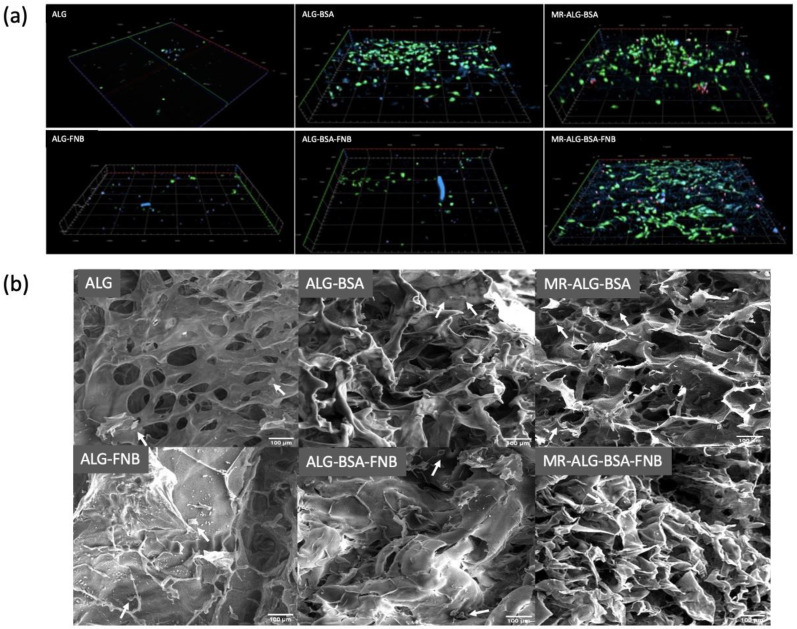
AREP-19 cells seeded on ALG-based scaffolds. (**a**) 3D reconstruction of confocal microscope images of seeded ARPE-19 cells on scaffolds with fluorescence staining after 14 days: DAPI (blue) calcein-AM (green) PI (red) in the depth of 200 µm of scaffolds (scale bar: 100 µm).; y = 1200 µm, x = 1200 µm, thickness = 200 µm, (**b**) SEM images of cells distributed on the ALG-based scaffolds; (Scale bar: 100 µm). White arrows indicate the presence of ARPRE-19 cells.

The confocal fluorescence microscopy images of stained cells in scaffolds (in the depth of 200 µm) after day 14 are presented in Figure 5a. To better understand cell deposition inside scaffolds, the 3D images were reconstructed. The DAPI showed more cells interacting with BSA-containing scaffolds than with ALG scaffolds. Cells seeded on ALG and ALG-FNB scaffolds showed minimal calcein-AM fluorescence, while more PI fluorescence was observed, indicating that cells could not expand nor distribute homogeneously within the scaffold’s matrix. In good agreement with the MTS assay, BSA-containing scaffolds showed more intense calcein-AM fluorescence, and reduction in the PI one, suggesting better cell–scaffold interactions and more viable cells within the scaffolds. This could be explained by the larger pore that the incorporation of BSA produced in the scaffolds, allowing deeper cell deposition and better transport of nutrients and oxygen. In addition, larger pores would be occluded later than smaller pores during progressive cell growth, and the presence of open spaces could keep the movement of oxygen and nutrients supply throughout the matrix [70].

The capacity of cells to homogenously distribute Inside the scaffold structure is essential to regenerate damaged tissue, as regions devoid of cells might lead to defective tissue structure in regenerated organs [71]. While ALG scaffolds showed a limited deep distribution of cells, a considerably deeper distribution of cells inside BSA-containing scaffolds could be observed. This might indicate the capacity of cells to spread out and populate a deeper scaffold matrix in the presence of BSA. The enhanced porosity associated with the incorporation of BSA in scaffolds formulation might help reduce cell aggregation at scaffolds surfaces which is essential for new tissue formation. In addition, SEM images of seeded cells on scaffolds, shown in Figure 5b, further demonstrated more cells present at the surface of ALG-BSA, MR-ALG-BSA, and MR-ALG-BSA-FNB scaffolds in comparison with ALG and ALG-FNB ones, providing a more favourable environment for cells.

## 4. Conclusions

This work was undertaken to design and develop different types of ALG-based scaffolds and evaluate their potential use for retinal regenerative and drug delivery applications. The potential role of BSA as a porogenic to enhance the porosity of ALG scaffolds was demonstrated. Furthermore, the incorporation of BSA in ALG scaffold increased their loading capacity toward a hydrophobic drug. On the other hand, the conjugation of BSA with ALG via the Maillard reaction improved the mechanical stability of scaffolds, which is essential for implantation at the back of the eye. It allowed a favourable swelling and sustained release of loaded FNB. In vitro biological investigations demonstrated the biodegradability and biocompatibility of BSA-containing ALG scaffolds, highlighting a significant enhancement of ARPE-19 cell viability distribution within the scaffolds when scaffolds were subjected to the Maillard reaction. Taken together, our results demonstrate that scaffolds made of ALG-BSA Maillard reaction products have a promising role for retinal tissue regenerative application and as an ocular drug delivery system.

## Data Availability

All data relevant to the publication are included.
